# Risk factors for a positive SARS-CoV-2 PCR in patients with common cold symptoms in a primary care setting – a retrospective analysis based on a joint documentation standard

**DOI:** 10.1186/s12875-020-01322-7

**Published:** 2020-12-03

**Authors:** Johannes Just, Marie-Therese Puth, Felix Regenold, Klaus Weckbecker, Markus Bleckwenn

**Affiliations:** 1 Department of General Practice and Interprofessional Care, Witten/Herdecke University, Alfred-Herrhausen-Straße 50, 58453 Witten, Germany; 2 Department of Medical Biometry, Informatics and Epidemiology (IMBIE), University Hospital Bonn, Sigmund-Freud-Str. 25, 53127 Bonn, Germany; 3 Department of General Practice, Leipzig University, Medical Faculty, Ph.-Rosenthal-Str. 55, 04103 Leipzig, Germany

**Keywords:** COVID-19, PCR, Positive-predictive value, point-of-care testing, risk factors

## Abstract

**Background:**

Combating the COVID-19 pandemic is a major challenge for health systems, citizens and policy makers worldwide. Early detection of affected patients within the large and heterogeneous group of patients with common cold symptoms is an important element of this effort, but often hindered by limited testing resources, false-negative test results and the lack of pathognomonic symptoms in COVID-19. Therefore, we aimed to identify anamnestic items with an increased/decreased odds ratio for a positive SARS-CoV-2 PCR (CovPCR) result in a primary care setting.

**Methods:**

We performed a multi-center cross-sectional cohort study on predictive clinical characteristics for a positive CovPCR over a period of 4 weeks in primary care patients in Germany.

**Results:**

In total, 374 patients in 14 primary care centers received CovPCR and were included in this analysis. The median age was 44.0 (IQR: 31.0–59.0) and a fraction of 10.7% (*n* = 40) tested positive for COVID-19. Patients who reported anosmia had a higher odds ratio (OR: 4.54; 95%-CI: 1.51–13.67) for a positive test result while patients with a sore throat had a lower OR (OR: 0.33; 95%-CI: 0.11–0.97). Furthermore, patients who had a first grade contact with an infected persons and showed symptoms themselves also had an increased OR for positive testing (OR: 5.16; 95% CI: 1.72–15.51). This correlation was also present when they themselves were still asymptomatic (OR: 12.55; 95% CI: 3.97–39.67).

**Conclusions:**

Several anamnestic criteria may be helpful to assess pre-test probability of COVID-19 in patients with common cold symptoms.

**Supplementary Information:**

**Supplementary information** accompanies this paper at 10.1186/s12875-020-01322-7.

## Background

The COVID-19 pandemic is a major challenge for health systems, citizens and policy makers worldwide [1]. The early political implementation of individual distancing has slowed down the reproduction rate of the virus in Germany significantly [2]. In addition, compared to other countries, Germany has implemented an intensive testing strategy for new infections using SARS-CoV-2 PCR (CovPCR), funded by the national social health insurance system (statutory health insurance). According to data from “Robert-Koch Institut” (RKI, “German CDC”) 176 participating laboratories reported a number of 348,619 PCR tests carried out in calendar week 12/2020 (419 tests/100.000 inhabitants). The tests were mostly carried out in doctors’ (GP’s) practices, newly formed COVID-19 test centers and hospitals [3].

In view of the potentially exponential increase in the number of new cases and finite laboratory resources (test stations, reagents, etc.), there is an urgent need to collect and evaluate clinical data on the clinical features of tested patients. Of particular interest is the question of how far patients with positive and negative results differ in their initial clinical presentation. The more precisely physicians use testing resources like point-of care COVID-19 antibody tests or Cov-PCR, the more efficient they are. Statistically, this is reflected in Bayes’ theorem: The quality of a test is not only determined by specificity and sensitivity, but also depends on the pre-test probability of the event to be tested. Thus, if the pre-test probability for COVID-19 is higher, the probability that a test-positive patient is actually ill (positive-predictive value = PPV) is increased and vice versa for negative CovPCR results (negative-predictive value = NPV) [4].

In this paper we aimed to identify predictive risk profiles for a positive CovPCR result in primary care.

## Method

### Study design

In this multi-center, cross-sectional study, the characteristics of patients who tested positive and negative for COVID-19 were assessed. A total of 26 office-based specialists for internal and/or general medicine with a full primary care mandate from 14 different locations participated in the study.

### Setting

All locations collected patient-related data based on a uniform quality standard in the documentation of COVID-19 suspect cases that we provided. Each site reported anonymous data on all CovPCR taken. All data were noted on a paper-based, structured documentation form and passed on anonymously for evaluation. The documentation form was prepared on the basis of the recommendations of the RKI as well as the available evidence on symptoms and risk factors for COVID-19 and was subjected to pre-testing with experienced family physicians [5–8].

### Indications for testing and test logistics

Indications for testing were based on the testing protocol that was issued by the RKI at the time as a strong recommendation and was adhered to by all participating physicians. The RKI testing recommendation was a web-based resource at the time and was changed several times since and therefore cannot be cited presently, but a screenshot is available from the authors upon request. It included the following criteria:

1. Acute respiratory symptoms AND Contact with a confirmed COVID-19 case
2. Acute respiratory symptoms AND signs of viral pneumonia
3. Acute respiratory symptoms AND health care worker
4. Acute respiratory symptoms AND risk factor for complicated course of disease
5. Acute respiratory symptoms AND free testing capacity
6. No acute respiratory symptoms but high risk contact with a confirmed COVID-19 case
7. None of the reasons mentioned above

(Indications 6 and 7 were not part of the RKI recommendation but reflected clinical testing reality and were therefore included in the questionnaire).

Within the German primary care system, general practitioners send PCR material to a qualified local laboratory for further testing. Usually, specimens are collected on a daily basis by the laboratory with which the general practitioner has chosen to cooperate. National quality management regulations apply to all laboratories performing PCR testing.

### Study period

The study period was from 24.03.2020 to 17.04.2020 - this period was chosen because the RKI indications for CovPCR remained unchanged and the COVID-19 incidence was high.

### Participants

The participating GP’s practices represented a “convenience sample”. Selection criteria were the performance of CovPCR, structured documentation and willingness to participate. All patients who received CovPCR in the participating GP’s practices within the study period were eligible for inclusion. Patients, whose tests had been carried out for procedural reasons and did not correspond to a specific clinical indication as specified above, were excluded (e.g. testing of recovered patients after end of quarantine). There were no other exclusion criteria.

### Variables and data sources

The variables under study included: Age, sex, reason for the test, risk factors, symptoms leading to the test, result expected by the GP and actual CovPCR results. All information was extracted from the anonymized documentation forms.

### Bias

In order to ensure that there was no excessive distortion in the patient collective due to the convenience sampling of practices, age and sex of the tested persons were compared with demographic data from a large national sample of CovPCR-tests [3].

### Statistical methods

Data entry was performed twice and the data sets were compared digitally, any deviations were checked and a plausibility check of the data entered was performed. The statistical evaluation was carried out with the statistics program R. In addition to the descriptive statistics, initial chi-square tests were performed for different potential influencing factors (symptoms, test reason, etc.) on the test result (CovPCR positive yes/no). Statistically significant influencing factors were then transferred to a logistic regression model to check their effect strength. Missing data were reported in the descriptive part, for the regression model missing values were assigned to the more frequent result (deviation to the middle), a proportion of missing values > 5% was not exceeded per variable. As there was some concern, that adding asymptomatic patients to the regression model may cause deviations to the outcome, a sensitivity analysis was performed and will be reported in the addendum.

The study design was reviewed by the ethics committee of the University of Leipzig, Germany under the procedure number 184/20-ek, there were no ethical concerns.

This study was conducted without external financial support.

## Results

The participating practices tested *n* = 374 patients per CovPCR in the investigated period. Of these, 10.7% (*n* = 40) tested positive. The symptom anosmia, as well as contact with infected persons, was associated with a positive CovPCR result.

A total of 26 specialists with a general practitioner mandate at 14 different locations (individual and group practices) from North Rhine-Westphalia, Rhineland-Palatinate, Hesse and Saxony-Anhalt (four of 16 federal German states) took part in the study. All practices are located in urban and rural districts with moderate to increased COVID-19 activity (cumulative incidence 50–250 cases per 100,000 inhabitants) [2].

**Fig. 1:**
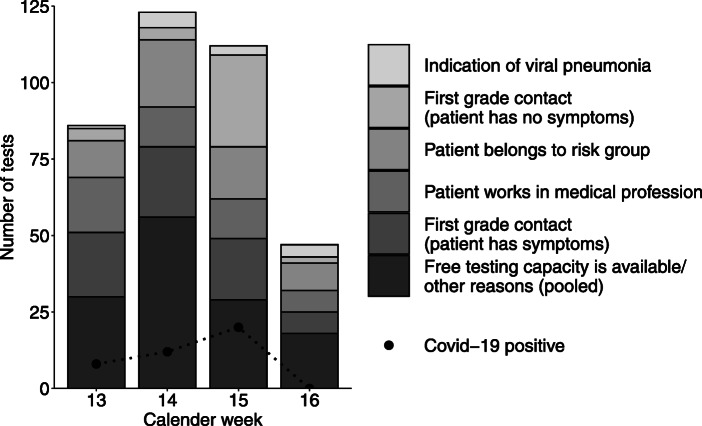
Number of tests separated by test reason per calendar week

Parallel to the number of new infections reported throughout Germany, the number of tests performed decreased in the course of the study, as did the rate of positively tested patients (Fig. 1).

Of the examined patients, 58.5% were female and the median age was 44 years. Further characteristics of the investigated cohort can be found in Table 1.

**Table 1:** Patient characteristics

	Total (*n* = 374)	COVID-19 negative (*n* = 334)	COVID-19 positive (*n* = 40)	*P*-value
Age				0.147*
MV (SD)	47.0 (20.2)	46.4 (19.7)	52.4 (23.5)	
Median (Q1-Q3)	44.0 (31.0–59.0)	43.5 (31.0–58.0)	52.0 (31.2–73.2)	
Missing, n (%)	4 (1.1)	4 (1.2)	0 (0.0)	
Gender, n (%)				0.475
Male	154 (41.2)	140 (41.9)	14 (35.0)	
Female	217 (58.0)	191 (57.2)	26 (65.0)	
Missing	3 (0.8)	3 (0.9)	0 (0.0)	
Reason for testing, n (%)				*< 0.001*
First grade contact (has symptoms)	72 (19.3)	58 (17.4)	14 (35.0)	
First grade contact (has no symptoms)	40 (10.7)	27 (8.1)	13 (32.5)	
Indication of viral pneumonia	13 (3.5)	12 (3.6)	1 (2.5)	
Works in medical profession	51 (13.6)	47 (14.1)	4 (10.0)	
Belongs to risk group	60 (16.0)	59 (17.7)	1 (2.5)	
Free capacity/other	134 (35.8)	127 (38.0)	7 (17.5)	
Missing	4 (1.1)	4 (12.0)	0 (0.0)	
GP expects positive result, n (%)				*0.010*
No	245 (65.5)	227 (68.0)	18 (45.0)	
Yes	119 (31.8)	99 (29.6)	20 (50.0)	
Missing	10 (2.7)	8 (2.4)	2 (5.0)	
Number of risk factors, n (%)				N/A
None	187 (50.0)	168 (50.3)	19 (47.5)	
One or two	138 (36.9)	125 (37.4)	13 (32.5)	
More than two	49 (13.1)	41 (12.3)	8 (20.0)	

*Mann-Whitney U Test

*MV* Mean value, *SD* Standard deviation, *Q1* 25%-quantile, *Q3* 75%-quantile

The most frequent symptoms at the presentation of patients who later tested positive for COVID-19 were cough, fever, anosmia and muscle pain. The most common symptoms at presentation of patients who later tested negative for COVID-19 were cough, sore throat, fatigue and fever. A complete list of symptoms is displayed in Table 2.

**Table 2:** Symptoms of patients tested negative and positive for COVID-19

Variable	COVID-19 negative (*n* = 285)		COVID-19 positive (*n* = 26)		*P*-value
Cough (n, %)	214	75.1	19	73.1	0.815
Sore throat (n, %)	120	42.1	5	19.2	0.023
Fatigue (n, %)	89	31.2	5	19.2	0.266
Fever (n, %)	84	29.5	9	34.6	0.655
Nasal congestion (n, %)	84	29.5	5	19.2	0.366
Muscle pain (n, %)	59	20.7	7	26.9	0.456
Dyspnea (n, %)	56	19.6	4	15.4	0.796
Headache (n, %)	47	16.5	3	11.5	0.780
Anorexia (n, %)	28	9.8	2	7.7	1.000
Diarrhea (n, %)	23	8.1	1	3.8	0.706
Anosmia (n, %)	22	7.7	7	26.9	0.006
Chills (n, %)	20	7.0	5	19.2	0.045
Nausea (n, %)	11	3.9	0	0.0	0.608
Vomiting (n, %)	4	1.4	0	0.0	1.000
Other symptoms (n, %)	37	13.0	3	11.5	1.000

Asymptomatic patients (*n* = 63) were excluded in this table

The regression model used to determine the individual effect sizes included all patient characteristics that showed a statistically significant influence on the test result in the Chi-square test. The odds ratio (OR) for a positive CovPCR was increased in patients who had contact with an infected person who were older and in patients with anosmia and decreased in patients suffering from a sore throat. Table 3 shows all results of the regression model. The sensitivity analysis performed excluding asymptomatic patients showed no meaningful changes in the ORs and CIs and was moved to the addendum for reference (Supplementary Table 4 - Addendum).

**Table 3:** Logistic regression model: factors influencing positive CovPCR

Variable	COVID-19 negative (*n* = 330) n (%)	COVID-19 positive (*n* = 40) n (%)	Adjusted OR (95% CI)	*P*-value
First grade contact (has symptoms)	58 (17.5)	14 (35.0)	5.16 (1.72–15.51)	*0.002*
First grade contact (has no symptoms)	27 (11.2)	13 (32.5)	12.55 (3.97–39.67)	*< 0.001*
Free capacity/other	125 (37.9)	7 (17.5)	1.50 (0.46–4.87)	0,497
age^a^	46,4 (19.7)	52,4 (23.5)	1.02 (1.00–1.04)	*0,019*
GP expects positive result	97 (29.4)	20 (50.0)	1.98 (0.90–4.38)	0.092
chills	20 (6.1)	5 (12.5)	2.80 (0.83–9.43)	0.117
Anosmia	22 (6.7)	7 (17.5)	4.54 (1.51–13.67)	*0.011*
sore throat	118 (35.8)	5 (12.5)	0.33 (0.11–0.97)	*0.029*

*OR* Odds ratio, *CI* Confidence intervals. Patients with missing age (*n* = 4) were excluded

^a^mean value (standard deviation); Nagelkerke R^2^ 0.265

## Discussion

### Main result

The main result of this work is the shown correlation between a positive CovPCR and the symptom anosmia as well as a confirmed contact with an infected person, regardless of whether the tested person themself had symptoms or not. These findings may be useful to improve NPV and PPV of testing procedures that are used in clinical practice, especially those with less accuracy than CovPCR (e.g. point-of care COVID-19 antibody tests).

### Strengths and limitations

In this work, predictive factors for a positive CovPCR were investigated for the first time. The evaluation was performed with a multicentric approach and was based on a common documentation standard. The selection of the practices was non-randomized, as a rapid implementation of the study was preferred to a lengthier randomized selection in respect to the pandemics’ rapid evolution. This may have resulted in a distortion of the investigated patient collective, although, no conspicuous deviations were found in comparison to the demographic data of all patients tested in Germany as published by the RKI; the age median in our population was 44 years (IQR: 31–59) compared to 42 years (IQR: 29–56) in a sample of + 1.000.000 patients tested in Germany [3]. Therefore, the results could be considered representative for patients in Germany. The most relevant limitation is the small sample size resulting in wide CIs and a connected uncertainty regarding the strength and direction of several effects. Still, this is the first study to show clinical risk factors for a positive CovPCR and its results are in line with the current evidence base [5–8].

### Number of patients tested and rates of positives

The proportion of test-positive patients in our population was 10.7%, which is slightly above the average of positive tests performed in Germany during this period (North Rhine-Westphalia (9.3%), Rhineland-Palatinate (9.6%), Hesse (11.8%)) [3]. The number of tests decreased towards the end of the investigation period, which parallels the decreasing number of new COVID-19 infections in Germany as well as the end of the pandemic cold season in early spring.

### Factors that were associated with a negative test result

Negative tests were associated with the symptom “sore throat”. This result is consistent with the low prevalence of the symptom “sore throat” in confirmed COVID-19 cases [6]. The correlation found between age and test results is marginal (younger people were tested negative more often) and may be explained by local outbreaks in nursing homes. This effect will most likely disappear due to diffusion processes during the course of the pandemic and may not be present in countries with a more homogenous spreading pattern.

### Factors that were associated with a positive test result

Positive tests were associated with confirmed contact. This was to be expected, but it was surprising that this also applied to asymptomatic patients. He et al. concluded that 45% of infections occur in the pre-symptomatic phase of the patient, i.e. the patient is still asymptomatic but already has large amounts of virus replicated in the throat and is highly contagious [9]. This result underlines the particular relevance of contact anamnesis for the test decision, but also the relevance of early isolation of potentially affected contacts.

Of particular clinical importance is the observed association between the symptom anosmia and a positive PCR result. Anosmia has already been described as a relevant COVID-19 symptom in other studies, but this is the first study to show that anosmia may be a useful discriminator between COVID-19 and other, endemic respiratory tract infections [8, 10, 11]. Since the examined patients mainly presented with fever, cough, muscle pain and other cold symptoms, which are common with endemic respiratory tract infections, anosmia can be an important indication of COVID-19. Although anosmia is a secondary symptom of a variety of viral respiratory infections, the incidence of anosmia without acute or chronic rhinitis in COVID-19 is noteworthy [8, 10–12]. However, a possible incorporation bias has to be discussed in this context, meaning that using anosmia as a main testing criterion would inevitably bring up anosmia as a risk factor over time. On the other hand, following the RKI testing criteria from March/April 2020, we assume the participating physicians mainly focused on respiratory symptoms. The RKI testing criteria did not mention anosmia in March/April 2020 because reports of anosmia as an important symptom were still very new and unconfirmed findings then. Therefore we conclude that an incorporation bias would be much more likely now, as anosmia has been added to the RKI testing criteria and its importance has become common knowledge to doctors and layman alike.

Reporting from our clinical experience with COVID-19, anosmia was sometimes so pronounced in COVID-19 patients that it was presented as the sole symptom upon presentation. Therefore, we recommend that patients are always asked for fever, cough, dyspnea and anosmia as part of the initial stratification process. In case of isolated anosmia in the absence of chronic or acute rhinosinusitis, we recommend a testing for COVID-19 infection, as already suggested in a French case series by Villalba et al. and others [8, 11, 13].

## Conclusion

We were able to identify several anamnestic criteria which showed a correlation with a positive Cov-PCR result (anosmia, first-grade contact with and without symptoms) as well as negative Cov-PCR result (sore throat). These findings may be used to increase the positive as well as negative predictive value of different types of testing procedures by adding clinical context. As an example, a patient with first-grade COVID-19 contact and anosmia who tests negative in a point-of-care antibody test for SARS-COV-2 may be considered false-negative by the attending physician and may therefore receive additional CovPCR testing before quarantine measures are being relaxed.

## Supplementary Information

**Additional file 1: Supplementary Table 4:** Association between positive COVID-19 PCR and different factors using logistic regression – alternative calculation excluding asymptomatic patients

## Data Availability

The datasets generated and analysed during the current study are available from the corresponding author upon reasonable request.

## Abbreviations

CovPCR: SARS-CoV-2 PCR
PPV: Positive-predictive value
NPV: Negative-predictive value
