# Supplementary material for: Risk factors for a positive SARS-CoV-2 PCR in patients with common cold symptoms in a primary care setting – a retrospective analysis based on a joint documentation standard
Source: BMC Fam Pract. 2020 Dec 3;21:251. doi: 10.1186/s12875-020-01322-7 (PMC7713668; doi:10.1186/s12875-020-01322-7)
Supplement: Supplementary file 1 — Additional file 1: Supplementary Table 4: Association between positive COVID-19 PCR and different factors using logistic regression – alternative calculation excluding asymptomatic patients [file 12875_2020_1322_MOESM1_ESM.docx]

Table 4: Association between positive COVID-19 PCR and different factors using logistic regression – alternative calculation excluding asymptomatic patients.

| Variable | COVID-19 negative  (n=283)  n (%) | COVID-19 positive  (n=26)  n (%) | adjusted  OR  (95% CI) |
| --- | --- | --- | --- |
| first grade contact | 58 (20.5) | 14 (53.8) | 5.22  (1.66-16.45) |
| free capacity/other | 109 (38.5) | 6 (23.1) | 1.54  (0.44-5.35) |
| age* | 46.0 (19.2) | 52.7 (24.0) | 1.03  (1.00-1.05) |
| GP expects positive result | 85 (30.0) | 16 (61.5) | 2.18  (0.85-5.61) |
| chills | 20 (7.1) | 5 (19.2) | 2.75  (0.81-9.33) |
| anosmia | 22 (7.8) | 7 (26.9) | 4.59  (1.49-14.16) |
| sore throat | 118 (41.7) | 5 (19.2) | 0.33  (0.11-1.00) |

Asymptomatic patients and patients with missing age (n=65) were excluded.

OR = odds ratio; CI = confidence intervals

*mean value (standard deviation); Nagelkerke R^2^ 0.247
